# Introduction of gloved hand to cage induces 22-kHz ultrasonic vocalizations in male albino rats

**DOI:** 10.1371/journal.pone.0278034

**Published:** 2022-11-18

**Authors:** Seo-Kyoung Hwang, Cheryl Tyszkiewicz, Melissa Dragon, Kimberly Navetta, Rebecca Ferreira, Chang-Ning Liu

**Affiliations:** 1 Comparative Medicine, Pfizer Worldwide R&D and Medical, Groton, Connecticut, United States of America; 2 Drug Safety Research and Development, Pfizer Worldwide R&D and Medical, Andover, Massachusetts, United States of America; Belgrade University Faculty of Medicine, SERBIA

## Abstract

Rodents emit ultrasonic vocalizations (USVs) above the human hearing threshold of ~ 20 kHz to communicate emotional states and to coordinate their social interactive behavior. Twenty-two kHz USVs emitted by adult rats have been reported in a variety of aversive social and behavioral situations. They occur not only under painful or restraining conditions but can also be evoked by gentle cutaneous touch or airflow. This study aimed to test if placement of a human hand in a cage can evoke 22-kHz USVs. It was found that 36% of the adult male Sprague-Dawley and 13% of the adult male Wistar Han rats emitted 22-kHz USVs when a gloved hand was introduced into the cages. Average vocalization onset latencies were 5.0 ± 4.4 s (Sprague-Dawley) and 7.4 ± 4.0 s (Wistar Han) and the USVs had a stable frequency (22 kHz) across the calls, ranging from 0.1 to 2.3 seconds in duration. Surprisingly, no 22-kHz USVs were found in any female Wistar Han rats tested. To further explore the mechanisms underlying this observation, we compared retinal function, basal serum corticosterone, and testosterone levels between the 22-kHz USV responders and non-responders. None of these parameters or endpoints showed any significant differences between the two cohorts. The results suggest that the introduction of a gloved-hand inside the cage can trigger adult male albino rats to emit 22-kHz ultrasonic vocalizations. This response should be considered in USV studies and animal welfare.

## Introduction

Murine rodents emit ultrasonic vocalizations (USVs) to socially communicate with each other and USVs reflect the physiological, motivational, and emotional states of animals [1, 2]. USVs have become a well-established measure of emotional/affective states in rodents [3]. In rats, frequency-modulated 50-kHz USVs have been thought to reflect a positive affective state [4], whereas flat (or solid line) 22-kHz USVs reflect a negative affective state [5]. Adult rats emit long-lasting 22-kHz USVs in a variety of aversive stressful social and behavioral situations, including agonistic encounters, social threats, air puffs, acoustic startle [6], presence of a predator [7], or other stressful behavioral situations (for more details see [1, 3]). Because of its close relation to aversive events, the 22-kHz USVs are often regarded as a correlate of fear or anxiety [6, 8–12] and often serves as an additional measure in fear conditioning experiments [13, 14]. In addition, the initial handling of rats also caused the production of ultrasonic vocalization in some rats [15]. More recently, one group found that even gentle touch in rats can cause 22-kHz USVs [16]. To the best of our knowledge, there is no study evaluating 22-kHz USV emissions triggered by the presence of a hand in cage. Whether introduction of a gloved hand could trigger rats to vocalize is unknown. Rodents collect external/environmental information via their sensory systems, such as visual, hearing, smell, taste, and touch/pain. In albino rats, spontaneous retinal degeneration has been reported [17]. The purpose of the present study was to test whether the introduction of a gloved human hand in the cage could evoke USVs in naïve albino rats with or without normal visual function.

## Material and methods

### Animals and study design

Groups of 14 male Sprague-Dawley (7–8 weeks) and 99 Wistar Han rats (48 males and 51 females, 16–20 weeks) rats were purchased from Charles River Laboratory (Raleigh, NC). All activities involving animals were carried out in accordance with federal, state, local, and institutional guidelines governing the use of laboratory animals in research and were reviewed and approved by Pfizer’s Institutional Animal Care and Use Committee. The animals were group-housed (2-3/cage) in Techniplast cages with Enrich-n’Pure bedding (The Andersons Inc., Maumee, OH) in an AAALAC-accredited vivarium with a room temperature of 20–26°C and humidity of 30–70 %, under a 12 h:12 h light-dark cycle, and had *ad libitum* water and regular irradiated Teklad Global Rodent Diet (Envigo, 2916C). Animals were acclimated to the environment for at least 5 days before experimental procedures were initiated.

### Experimental setup and ultrasonic vocalization recording

**Fig 1:**
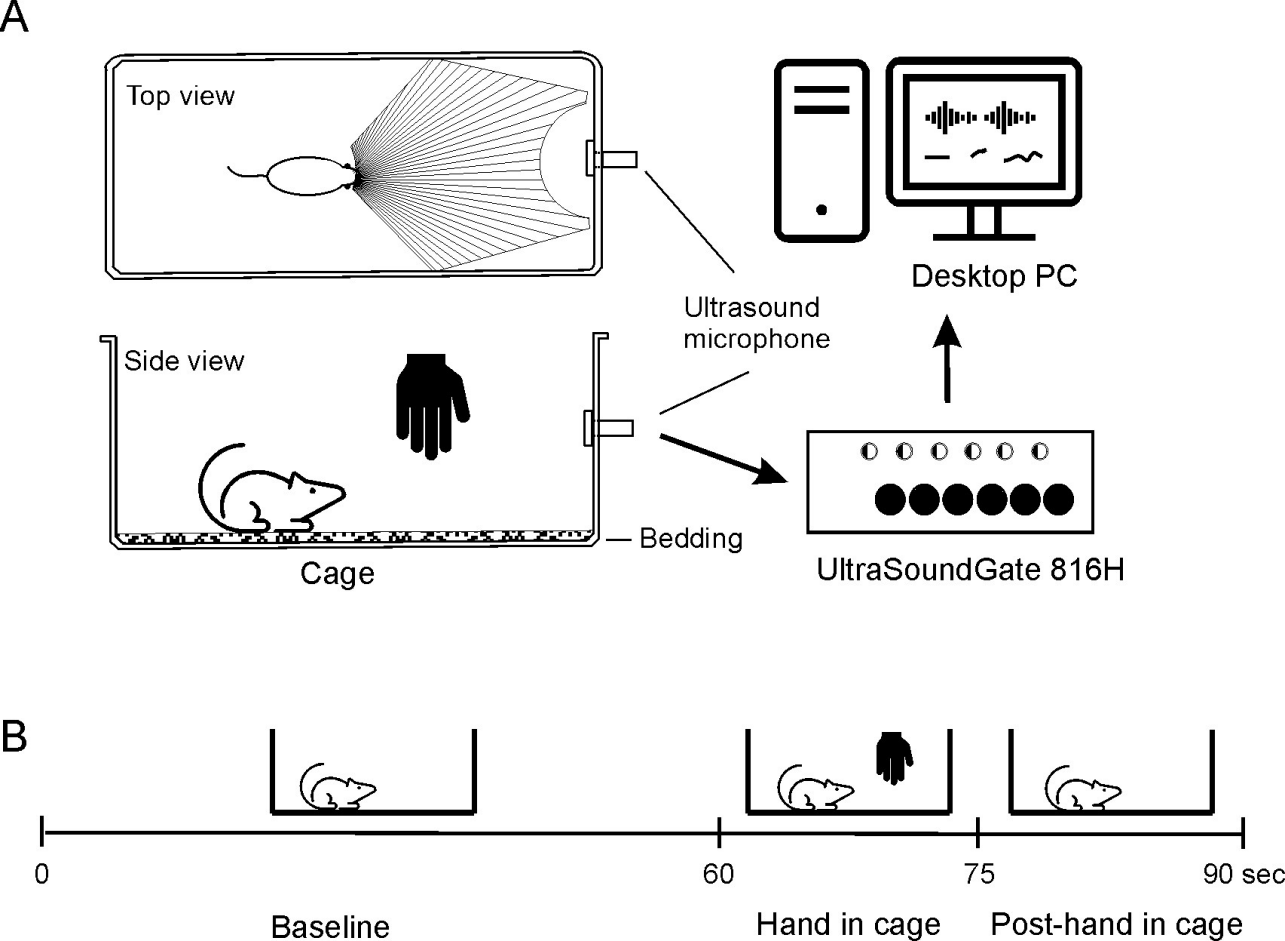
The USV experimental cage and set up. (A) The floor of the animal chamber was disinfected and covered with a thin layer of fresh bedding. The ceiling of the cage was removed for easy hand movement during the test. The tester’s hand with a glove was placed in the marked area (top view) where the animal can see. If animal moved and the hand was out of the shaded range, the hand followed and then held still accordingly to keep the hand in front of the face. (B) Test phases.

One rat was tested at a time. During the two successive test days, the animals were moved from their home cages to a semi-transparent polycarbonate test cage measuring 23 cm width × 34.5 cm length × 19.5 cm high without a lid. To avoid potential olfactory cues to animals, the bedding was changed between each animal if they came from different home cages. In the test cage, an ultrasound microphone was inserted and fixed in the center of the short wall to capture USV signal emitted by a rat. The ceiling of the cage was removed for easy hand movement during the test. During the test, the tester’s hand with a glove was placed in front of the animals where the animal can see. If an animal moved out of the range, the hand followed and held still accordingly for 15 seconds. The emission of USVs by the subject animal was captured by an UltraSoundGate condenser ultrasonic microphone (CM16, Avisoft Bioacoustics, Berlin, Germany), sensitive to frequencies between 15 and 180 kHz (flat frequency response between 25 and 140 kHz; ± 6 dB). This apparatus was mounted in the center of the short wall of the cage and connected to a computer via an UltraSoundGate IH8 (Avisoft Bioacoustics, Fig 1A). Acoustic data were recorded by Avisoft Recorder software (version 2.95, Avisoft Bioacoustics), using a sampling rate of 250,000 Hz in 16-bit format and a recording range of 0–125 kHz.

Experiments were conducted in a room with 250 lx illumination at the animal’s eye level. After a 1-minute baseline period, a hand with a glove (Microflex SuprenoEC, Ansell) was inserted and held in the cage in front of the animal’s visual field (shadowed area in Fig 1A) for 15 sec. Fifteen seconds after the gloved-hand was removed the USV recording was completed, and the animal was removed and returned to its home cage (Fig 1B). For offline analysis of the acoustic data, SASLab Pro software (version 5.3) was used. After a fast Fourier transformation (256 FFT length, 100% frame, FlatTop window, and 50% time window overlap), high-resolution spectrograms were produced with a frequency resolution of 586 Hz and a time resolution of 0.8533 ms. The following parameters were determined and calculated: latency of the first call from the animal “seeing” the gloved-hand, number of calls during the 15-sec test phase, mean call duration, mean peak frequency, and type of the calls. The separation between one call and another was defined by an inter-call interval of at least 190–320 ms, which is the time for inhalation between two calls [18].

### Electroretinography (ERG)

Full-field flash electroretinography (ERG) was performed in a subset of rats from both strains as described previously [19]. In brief, under anesthesia with isoflurane at 2–3% mixture of oxygen, the animals were placed on a heating pad on a platform and the body temperature was monitored through a rectal thermometer and maintained at ~36–37°C. Pupils were dilated using tropicamide (1%, Akorn, Lake Forest, IL). A drop of GenTeal tears (Alcon, Geneva, Switzerland) was applied on the cornea to prevent its dehydration and allow electrical contact with the recording electrode. A 25-gauge platinum needle electrode was inserted under the scalp, between the two ears, and served as the reference electrode. An UTAS BigShot Visual Electrodiagnostics System (LKC Technologies, Inc., Gaithersburg, MD) was used to evoke and acquire the ERG signals (high–pass filtered at 0.3 Hz and low–pass filtered at 500 Hz). ERG protocols were adapted from Rosolen et al. [20] to test photopic luminance responses of the retina. The photopic responses were obtained with the background Ganzfeld illumination of 30 cd /m^2^ (white light, generated by the BigShot system and calibrated by LKC Inc.). The responses were tested at the flash intensities of 0.023–7.8 cd-s/m^2^. ERG waveforms were analyzed with LKC Technologies provided software using the International Society for Clinical Electrophysiology of Vision (ISCEV) guidelines. *b*-Wave amplitude was measured from baseline to b-wave peak, and *b*-wave latency was measured from stimulus to *b*-wave peak.

### Corticosterone and testosterone measurements

Blood was collected via cardiac puncture from a subset of male non-responders and all responders after euthanasia with CO_2_ on 20 days following the USV test. Serum corticosterone and testosterone were quantitated using two separate ultra-high-pressure liquid chromatography-tandem mass spectrometry assays performed on a classic waters ACQUITY UPLC system (Milford, MA) with an AB Sciex 6500+ Qtrap (Framingham, MA) developed in house for rat samples and are similar to those published in the literature [21–23]. In brief, prior to use, each assay was tested for intra- and inter-assay precision, accuracy, and carry-over. Testosterone samples were prepared for analysis by the addition of an internal standard followed by solid-phase extraction. Corticosterone samples were prepared for analysis by the addition of an internal standard followed by protein precipitation. Corticosterone and testosterone were separated from other sample components by gradient elution with reverse phase chromatography and then detected by monitoring unique fragment ions in the mass spectrometer. Calibration curves for corticosterone and testosterone were constructed from known standards by plotting the ratio of the analyte peak to internal standard peak area versus concentration. The concentration in the samples was calculated by interpolation using 1/x^2^ weighted linear regression analysis from the calibration curve for each analyte. Assay performance was determined by quality control standards throughout each individual run.

### Statistical analyses

All data were expressed as mean ± standard deviation (SD). The student’s 2-tailed t-test was used to compare the parameter between responders and non-responders with P = 0.05 as a significant threshold.

## Results

### Introduction of gloved hand into cage induces 22-kHz and 50-kHz ultrasonic vocalizations in SD and WH rats

**Fig 2:**
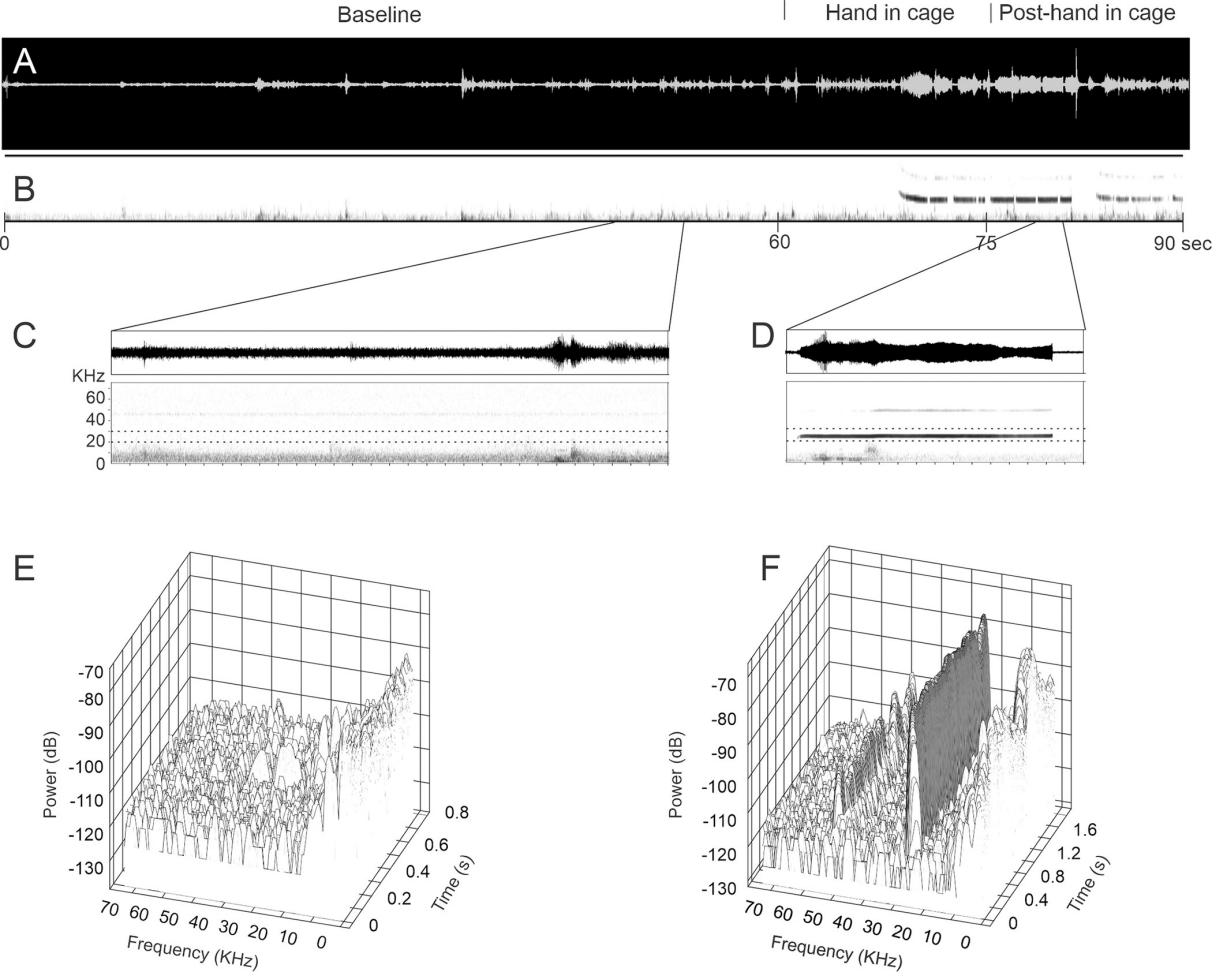
Representative spectrograms of 22-kHz USVs emitted by an adult SD rat. (A) the waveform of the vocalization signals (in voltage) captured during the baseline, hand-in-cage, and hand-out phases. (B) short sections of the spectrogram of the above signals show a typical sequence expanded in the X-axis in the baseline phase (C) and hand-in cage phase (D). Note the 22-kHz USVs signals in (D) but absent in (C). (E) and (F) show 3-dimensional graphs of USVs as shown in (C) and (D), respectively. Note the wall-like distribution of the 22-kHz USV signals in hand in cage phase (F) but not in baseline phase (E).

In 14 male SD rats tested, there were no spontaneous 22-kHz USVs in the baseline phase prior to hand introduction in the cage. Five rats (36%, Table 1) showed vocalization during the phase when a hand was placed in the cage for 15 sec. After a mean latency of 5 ± 4. 4 s, 33 ± 6 (mean ± SD) calls were evoked by the hand with a steady frequency of around 22 kHz, and the average duration of the calls was 0.65 ± 0.31 s (ranged from 0.1–1.5 s). Similarly, among 48 male WH rats tested, 6 rats (12.5%, Table 1) showed vocalization during the hand-in-cage phase (one WH animal had spontaneous 22 kHz USVs in the baseline phase prior to the introduction of gloved hand). After a mean latency of 8.2 ± 4.3 s, 21 ± 1.9 calls were evoked by the hand with a steady frequency of about 22 kHz, and the average duration of the calls was 0.95 ± 0.55 s (ranged from 0.2–2.3 s, Table 2). When the hand was moved from the cage (post-hand in the cage), Animals still emitted after-discharge USVs over the following 15 secs (Table 2, Fig 2). Surprisingly, none of the female WH rats emitted 22-kHz signals.

**Table 1:** Prevalence of hand-induced 22-kHz ultrasonic vocalizations in Sprague-Dawley and Wistar Han rats.

Sex	Strains	N of responders	N of non-responders	Prevalence (%)
**Male**	Sprague-Dawley	5	9	36
	Wistar Han	6	42	12.5
**Female**	Wistar Han	0	51	0

**Table 2:** Characteristics of hand-induced 22-kHz vocalizations in male SD and WH rats (mean ± SD).

Strains (n)	Latency (second)	Number of calls (hand-in-cage phase)	Duration (second)	Number of calls after hand phase
**Sprague-Dawley (5)**	5.0 ± 4.4	33 ± 5.9	0.65 ± 0.31	46.5 ± 17.3
**Wistar Han (6)**	8.2 ± 4.3	21 ± 1.9	0.95 ± 0.55	27.5 ± 2.6

We also analyzed 50-kHz responses in these animals. There seems to be gender difference in 50-kHz USVs emission when introducing a gloved hand in the cage. We found that 39% of females significantly emitted ~50 kHz USVs when a gloved hand was introduced (S1 Table), which was different from 22 kHz USV emissions as stated above. Interestingly, we did not observe that 22 kHz USV emissions were interspersed with 50 kHz calls in the same male SD or WH rats. In addition, females showed more attractive behaviors to the gloved hand than males, e.g., more frequent touching behavior to the gloved hand.

### Electroretinography (ERG) comparison between the 22-kHz responders and non-responders

To explore whether USVs responders and non-responders had a similar visual function, standard full-field flash ERG was performed in the male animals under the similar light (photopic) condition as the light in the animal room. There were no differences between responders vs. non-responders in the amplitudes or latencies of ERG *b*-wave tested at 4.15 or 7.80 cd-s/m^2^ (Table 3). Interestingly, two WH responders had an abnormal ERG waveform. The *b*-wave was virtually undetectable for any light stimuli applied up to 7.8 cd-s/m^2^, which implies that these animals had poor or impaired vision.

**Table 3:** ERG parameter comparison (at maximum intensities of 5 dB) between male 22-kHz responders and non-responders.

Strains	Animal	*b*-wave latency	*b*-wave amplitude
		(ms, [n])	(μV, [n])
**Sprague-Dawley**	Responders	45.18 ± 6.43 [5]	87.94 ± 53.47 [5]
	Non-responders	42.24 ± 2.76 [9]	104.62 ± 46.37 [9]
**Wistar Han**	Responders	47.53 ± 2.90 [4*]	106.9 ± 7.28 [4*]
	Non-responders	45.18 ± 0.72 [35]	115.3 ± 5.41 [35]

Values are mean ± SD

*: Of the 6 responders, 2 of them had abnormal ERGs and were therefore excluded/ unable to analyze.

### Corticosterone and testosterone comparison between the 22-kHz responders and non-responders

Since stressful or fearful behaviors are related to hormones, we compared corticosterone and testosterone levels between the responders and non-responders. There was no statistically significant difference in either hormone between the two cohorts (Table 4).

**Table 4:** Corticosterone and testosterone concentration comparison between 22-kHz responders and non-responders.

Strains	Animal	Corticosterone	Testosterone
	(n)	(ng/mL)	(ng/mL)
Sprague-Dawley	Responders (5)	74.86 ± 19.18	146.7 ± 57.48
	Non-responders (9)	80.62 ± 32.28	150.7 ± 66.38
Wistar Han	Responders (6)	109.7 ± 47.41	140.2 ± 86.44
	Non-responders (6)	94.97 ± 57.47	288.3 ± 154.7

## Discussion

Acclimation to handling is important to reduce or eliminate fear/stress before potential behavioral assessments. Also from an animal use and care perspective, initial handling/acclimation upon arrival/at a young age might improve quality of life in long-duration studies and for colony animals. To the best of our knowledge, the present study demonstrates for the first time that the 22-kHz USVs could be induced by a gloved-hand placed before the rats’ eyes in a cage. There was only one WH rat who had spontaneous 22-kHz USV calling prior to gloved hand introduction. Given its very low incidence (1/62, 0.2%), we considered it an outlier (due to native or other unknown reasons), and does not overturn our conclusion. The duration of the calls (0.1–2.3 sec) was in the same range as those reported when rats were touched (0.3–1.2 s) [16]. In a previous study, it was also found that rats exposed to a cat under semi-natural conditions emit 22-kHz USVs. Additionally, when even a control (toy cat) stimulus was presented, rats showed short-lived 22-kHz USVs [7]. Our current study implies that the rats might consider the gloved hand as an aversive cue. The pattern is also similar to the previous studies applying the hand touch or predator to rats [7, 16]. In adult rats, 50-kHz USVs have been well documented and suggested as an indicator of positive or affective states [3, 24]. It is frequently elicited by the psychostimulant (eg. amphetamine) or increased sexual motivation or during sexual encounters [25]. In our study, we observed 50-kHz USV emissions in some female animals (see S1 Table). However, since there is not much literature to support that the hand (except for tickling) can induce an affective response, we focused on 22-kHz USVs in this manuscript.

In our current study, the gloved hand evoked 22-kHz activities in a fraction of the animals tested. This brings up the question ‘why other animals did not respond with 22-kHz USVs to the same stimuli?’ Is this due to the possible inherited retinal dystrophy or deficit as reported, especially in female rats [17, 26]? To increase our understanding of the mechanism of the hand-induced USVs, we first checked the retinal function of all 22-kHz responders and all (SD) and subset (WH) non-responder animals to the photopic test stimuli, using the full-field flash ERG technique. It was notable that the photopic responses (*b*-wave amplitude and latency) of the non-responders were not different from that of responders in both strains (Table 3). Interestingly, two responders (Wistar Han strain) had abnormal or virtually no ERG signals when tested with the same light flash stimulation protocols. We argue that a gloved hand-induced 22-kHz USVs may also be mediated by non-imaging forming photoreceptors in the retina of rats, such as the ipRGC–suprachiasmatic nucleus sensory pathway [27]. Other possible mechanisms include the odor of gloves or gentle air movement caused by the initial brief hand movement. It is reported that male mice emit more USV syllables when exposed to freshly collected female urine, but not to frozen ones [28]. We speculate that the odor from the glove may not be strong enough to induce USVs, since it was reported that odor-shock induced a significant decrease in USVs in adult Long Evans rats [29]. The latter is less possible since a strong air-puff (at least at a pressure of 9–16 psi) was required to induce 22-kHz USVs [10]. Nonetheless, care must be taken in specifying exactly how the hand triggers the neurocircuits in the brain and finally evokes USV. It is well-documented that 22-kHz USVs are animals’ responses to stress [30, 31]. In animals’ stress systems, the hypothalamic-pituitary-adrenal (HPA) axis plays an important role. These glucocorticoids (also called stress hormones) represent the final effectors of the HPA axis, and in turn regulate the release of corticotropin-releasing factor (CRF) and adrenocorticotropic hormone (ACTH), via multiple negative feedback mechanisms. Rasmussen and co-workers found that a cage change influences serum corticosterone and anxiety-like behaviors in mice [32]. The elevation (about 25%) in plasma level of corticosterone has also been reported in male albino rats after exposure to an acute noise stressor [33, 34]. Our observation of 22-kHz USV emissions in response to a gloved hand in the cage prompted us to further investigate whether 22-kHz USVs are correlated with basal stress hormones. Therefore, we subsequently compared the blood corticosterone concentrations between the responders and non-responders. Unexpectedly, no significant differences in the concentration of corticosterone were found between the two groups (Table 4). Ideally, the blood sampling should be made concurrently when the hand is present in cage, which is very difficult without interfering with or contaminating USV signals. The other system responsible for the stress response is the autonomic nervous system (especially the sympathetic branches). In future experiments, it would be interesting to compare the neuronal structure or activities in the relevant brain areas, eg, the basolateral amygdala [35, 36], perirhinal cortex [37], or hippocampus [38], etc. One weakness of this study is that the WH rats were significantly older than the SD rats. While the USVs are quite different between infant and adult rats, to the best of our knowledge, there are no reported age-related changes in USV call counts and other parameters from weeks 1–24 [39]. Other investigators also use 24-week-old rats in their USV study [40]. With what has been reported in the literature, we believe there is still merit to test younger WH rats in the future when the animals are available.

It is curious that in this study, the USVs responses were only seen in males, in line with the hypothesis proposed in the literature that the production of 22-kHz USVs differs between sexes. For instance, in previous studies applying fear conditioning protocol, 62% of males with Wistar Han genetic background vocalized, but only 28% of females emitted 22-kHz USVs [41]. Whereas in Long-Evans rats, female rats show more frequent 22-kHz USVs than males when exposed to a cat [42]. In addition, in castrated mice, testosterone administration has been found to restore reduced USVs calling [43]. Similarly, in response to an air-puff, castrated male Wistar rats produced a shorter overall duration of alarm USVs than sham-operated or castrated male rats with a testosterone implant [44]. Elevation in serum levels of testosterone has also been reported in adult male albino rats after restraining stress [45] or handling [32, 46]. While it is technically challenging to measure these hormone changes in real-time vocalization, we further measured the basal level of testosterone and compared if the responders have higher baseline concentrations of this stress hormone. Different from our hypothesis, no significant difference in the basal testosterone, like corticosterone, was noticed between the responders and non-responders (Table 4). While we did not find any difference in the stress or sex hormones between the responders and non-responders in this study with limited animals, blood sampling from a larger population of animals for hormone quantification or other neurological structures, relevant neurotransmitters [47, 48], or their transporter/receptors [41] warrant further investigation.

In summary, our results clearly indicate the importance of caging and handling procedures in behavioral studies on adult rats. The gloved human hand might be considered at first by rats as a potential danger or threat. The animals also convey this vocal message to others via USVs. The present report on the vocalization response to a gloved hand suggests careful handling in the cage might be considered for animal welfare and psychophysiological experiments.

## Supporting information

S1 Table Prevalence of hand-induced 50-kHz ultrasonic vocalization in Sprague-Dawley and Wistar Han rats. (DOCX)
