# Supplementary material for: Introduction of gloved hand to cage induces 22-kHz ultrasonic vocalizations in male albino rats
Source: PLoS One. 2022 Nov 18;17(11):e0278034. doi: 10.1371/journal.pone.0278034 (PMC9674133; doi:10.1371/journal.pone.0278034)
Supplement: S1 Table — (DOCX) [file pone.0278034.s001.docx]

**S1 Table. Prevalence of hand-induced 50-kHz ultrasonic vocalization in Sprague-Dawley and Wistar Han rats**

| **Sex** | **Strain** | **N of responders** | **N of non-responders** | **Total incidence for 15 seconds** | **Prevalence**  **(%)** |
| --- | --- | --- | --- | --- | --- |
| **Male** | Sprague-Dawley  (n=14) | 0 | 14 | 0 | 0 |
|  | Wistar Han  (n=48) | 3 | 45 | 10 | 6.3 |
| **Female** | Wistar Han  (n=51) | 20 | 31 | 347 | 39.2 |
